# Phosphoglycerate dehydrogenase and Alzheimer’s disease

**DOI:** 10.17179/excli2025-8178

**Published:** 2025-03-05

**Authors:** Quan-Ying Liu, Shuang-Qing Zhang

**Affiliations:** 1 National Institute for Nutrition and Health, Chinese Center for Disease Control and Prevention, 27 Nanwei Road, Beijing 100050, China

## ⁯

As a prominent public health issue, people with Alzheimer's disease (AD) are estimated to increase from 55 million in 2019 to 139 million in 2050 with expected total cost of $ 14 trillion (Chen et al., 2024[1]). Cognition-related synaptic plasticity dysfunction is observed in the early stage of AD before prominent accumulation of amyloid plaques and neurodegeneration (Navakkode et al., 2021[7]). Phosphoglycerate dehydrogenase (Phgdh) is a key enzyme for the *de novo* synthesis of brain serine required for the modulation of synaptic plasticity via N-methyl-D-aspartate receptor (NMDAR) (Li et al., 2024[5]). Therefore, it is important for the evaluation of Phgdh expression in AD although changes in serine level have not been definitively associated with AD due to the controversial direction of changes of brain Phgdh expression level during the progression of AD. 

Le Douce et al. (2020[4]) found that 6-month-old female triple-transgenic AD model mice (3xTg-AD mice) did not present significant reduction of hippocampal Phgdh expression in comparison with age-matched controls, as well as a dramatic decline of brain Phgdh levels in intermediate AD and advanced AD patients of 62 % and 82 %, respectively. Unfortunately, they did not provide an accurate explanation for Phgdh alteration. Chen et al. (2022[2]) inferred that the sensitivity of human Phgdh protein to protease cleavage at room temperature possibly contributed to the decrease of Phgdh levels in Le Douce et al.'s postmortem human AD samples. In contrast to the results of Le Douce et al. (2020[4]), Chen et al. (2022[2]) claimed that both 12-month-old 3xTg-AD mice and 10-month-old human P301S tau transgenic mice (PS19) expressed significantly higher hippocampal Phgdh than littermate wild control, as well as no, early, and late AD subjects displayed sequential increase of hippocampal Phgdh expression. They supposed that there was a positive feedback loop between neural excitotoxicity and elevated astrocyte Phgdh expression. Plasma and brain Phgdh in older AD patients significantly increased as compared to age-matched controls (Yan et al., 2020[9]). These reports indicated that Phgdh expression as a biomarker in AD needed to be further investigated.

Phgdh deficiency-induced reduction of glutathione synthesis impairs the scavenging of intracellular reactive oxygen species (ROS), leading to the promotion of mitochondrial biogenesis in inflammatory macrophages (Wang et al., 2024[8]). Additionally, according to the neuroinflammatory pathogenetic hypothesis on AD, for microglia with similar functions to macrophages in the central nervous system, its activation plays a protective role in the early AD, however, its overactivation results in massive release of pro-inflammatory factors that promote the AD development (Chounchay et al., 2020[3]; Merighi et al., 2022[6]). Based on the evidence mentioned above, microglia overactivation and neuroinflammation fully support the reduction of Phgdh expression in the late AD. Therefore, in our opinion, the decrease of Phgdh levels in the late AD is more reasonable.

In conclusion, Phgdh represents a promising therapeutic target for AD. Further studies should focus on the relationship between Phgdh expression and neuroinflammation.

## Declaration

### Acknowledgments

None.

### Declaration of interests 

The authors declare no competing interests.

### Author Contributions

Quan-Ying Liu: original draft preparation. Shuang-Qing Zhang: conceptualization, supervision, review and editing. Both authors read and approved the final manuscript.
